# The Folding Landscapes of Human Telomeric RNA and DNA G‐Quadruplexes are Markedly Different

**DOI:** 10.1002/anie.202100280

**Published:** 2021-04-06

**Authors:** Diana Müller, Irene Bessi, Christian Richter, Harald Schwalbe

**Affiliations:** ^1^ Goethe University Frankfurt/Centre for Biomolecular Magnetic Resonance (BMRZ) Institute for Organic Chemistry and Chemical Biology Max-von-Laue-Str. 7 60438 Frankfurt am Main Germany; ^2^ Present address: Julius-Maximilians-University Würzburg Institute of Organic Chemistry Am Hubland 16 97074 Würzburg Germany

**Keywords:** folding landscapes, G-quadruplexes, kinetics, real-time NMR spectroscopy, TERRA RNA

## Abstract

We investigated the folding kinetics of G‐quadruplex (G4) structures by comparing the K^+^‐induced folding of an RNA G4 derived from the human telomeric repeat‐containing RNA (TERRA25) with a sequence homologous DNA G4 (wtTel25) using CD spectroscopy and real‐time NMR spectroscopy. While DNA G4 folding is biphasic, reveals kinetic partitioning and involves kinetically favoured off‐pathway intermediates, RNA G4 folding is faster and monophasic. The differences in kinetics are correlated to the differences in the folded conformations of RNA vs. DNA G4s, in particular with regard to the conformation around the glycosidic torsion angle *χ* that uniformly adopts *anti* conformations for RNA G4s and both, *syn* and *anti* conformation for DNA G4s. Modified DNA G4s with ^19^F bound to C2′ in arabino configuration adopt exclusively *anti* conformations for *χ*. These fluoro‐modified DNA (antiTel25) reveal faster folding kinetics and monomorphic conformations similar to RNA G4s, suggesting the correlation between folding kinetics and pathways with differences in *χ* angle preferences in DNA and RNA, respectively.

## Graphical abstract

We compare K^+^‐induced folding of an RNA G‐quadruplex (G4) with a sequence homologous DNA G4 using CD spectroscopy and real‐time NMR spectroscopy. The folding kinetics of RNA and DNA G4 are markedly different: while DNA folding is biphasic, shows kinetic partitioning and involves kinetically favoured off‐pathway intermediates, RNA folding is faster and monophasic. We attribute the differences to different stabilities of the torsion angle *χ*.

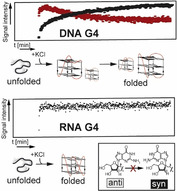

## Introduction

G‐quadruplexes (G4s) are four‐stranded oligonucleotides formed both by RNA and DNA G‐rich sequences that are stabilised through Hoogsteen hydrogen‐bonds and binding to monovalent cations, in particular K^+^. G4‐forming sequences are abundant in the human genome and their roles in gene regulation have been widely demonstrated.^[1]^ Due to their location in telomeres and in promoter‐regions of oncogenes, they have become increasingly important as potential drug targets.^[2]^

**Scheme 1:**
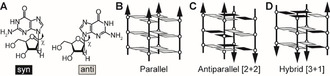
A) The glycosidic torsion angle *χ* can adopt either *syn* or *anti* conformation. B–D) Overview of possible strand orientations: B) parallel, C) antiparallel and D) hybrid [3‐1]. Grey filled or black filled rectangles indicate guanosines in *anti* or *syn* conformation, respectively.

DNA G4s are polymorphic and their structures differ in terms of strand orientation, conformation of the glycosidic torsion angle *χ* (*syn*, *χ*=40–80°, or *anti*, *χ*=180–240°) (Scheme 1) and geometry of loops connecting the G‐rich tracts.^[3]^

Previously, we have shown that the K^+^‐induced folding of human telomeric DNA G4s undergoes kinetic partitioning and long‐lived intermediates are populated.^[4]^ Insight from these experimentally observed K^+^‐induced folding investigations is supported by MD simulation.^[5]^

For a kinetic partitioning folding mechanism, a bifurcation of the folding pathway is characteristic for DNA G4. A kinetically favoured long‐lived intermediate is formed that first has to unfold before adopting the thermodynamically most stable state. Kinetically favoured and multiple thermodynamically stable states are populated, in line with the structural polymorphism of DNA G4s at equilibrium.^[4]^

Comparatively little is known about the kinetics and folding pathway of RNA G4, even if RNA G4s have first been observed already in 1991.^[6]^ RNA G4s have been identified in 5′‐UTRs,^[7]^ 3′‐UTRs,^[8]^ in introns^[9]^ as well as transcriptome of the telomeres (telomeric repeat‐containing RNA^[10]^ short: TERRA). In vivo studies linked translation regulation,^[11]^ splicing regulation^[12]^ and chromatin remodelling^[13]^ to the existence of RNA G4s. However, their existence in vivo is still matter of debate, but growing evidence has recently been reported for various G4 RNA functional roles.[^8^, ^14^]

In contrast to DNA G4s, RNA G4s are monomorphic irrespective of salt‐condition, molecular crowding or capping structures. Most structures determined thus far (pdb‐codes: 3MIJ, 2M18, 2KBP, 2RQJ, 6GE1, 6K84 6JJH, 4XK0) reveal RNA G4s to adopt an all‐*anti* all‐parallel structure (Scheme 1 B).^[15]^ The reason has been debated but not finally understood.^[16]^ Only in case of G‐rich aptamers as spinach and mango, exceptions have also been published.^[17]^ They contain discontinuous G‐stretches and long loops that can influence the G4 structure by formation of further stabilising secondary structures.

The investigation reported here were motivated by the hypothesis that RNA G4 folding should be faster, since the change from the more stable *anti* conformation for unfolded guanosine nucleotides to the less stable *syn* conformation adopted in antiparallel or hybrid G4 architectures would not be required. Further, the folding should be monophasic, since then intermediates on the RNA G4 folding pathway are not expected.

By application of time‐resolved NMR following K^+^‐induced folding of RNA G4, we indeed show here that the folding landscapes of human telomeric DNA and RNA G4 are fundamentally different. The kinetics of RNA G4 folding is in fact faster than DNA G4 folding kinetics. By comparison with ^19^F‐modified DNA in arabino configuration that also adopts *anti* conformations, we provide evidence that these altered folding kinetics are linked to the conformation around the glycosidic bond angle *χ*.

## Results and Discussion

We analysed the folding kinetics of human telomeric DNA (TA(GGGTTA)_3_GGGTT wtTel25) and TERRA RNA (UA(GGGUUA)_3_GGGUU TERRA25) G4s applying time‐resolved NMR spectroscopy and introduced ^19^F‐modifications to modulate the structure and the folding kinetics of DNA G4. From temperature‐dependent measurements, we determined thermodynamic parameters (*T*_m_, Δ*H*°, Δ*S*°, Δ*G*°) of all studied G4s.

### Folding Kinetics of wtTel25 and TERRA25 G4s

G4s are not only stabilised by Hoogsteen hydrogen bonds but also by monovalent cations including K^+^ and Na^+^ which are essential for G4‐formation. Therefore, G4 folding can be induced by addition of KCl and the build‐up of NMR‐signals resonating at ≈11.5 ppm characteristic for Hoogsteen base‐pairs can be analysed. A careful sample preparation is required to avoid G4‐promoting cations beforehand. A rapid mixing device^[18]^ was used to inject a KCl‐solution in situ into the NMR tube and folding experiments were conducted in a temperature range between 283 K and 298 K for three of the four systems. Due to a poor signal‐to‐noise (S/N) of TERRA25’s kinetic traces at 298 K (Figure S1), however, only the measurements at 283 K can be faithfully analysed. Previously, we investigated the folding kinetics of DNA G4s Tel24 (TT(GGGTTA)_3_GGGA) and other related DNA sequences with different flanking nucleotides. The DNA G4 folding kinetics is always biphasic and involves the formation of a long‐lived kinetic conformation. The nature of the flanking nucleotides determines the folding rate and the population ratio of major to minor conformation at equilibrium.^[4]^

**Figure 1:**
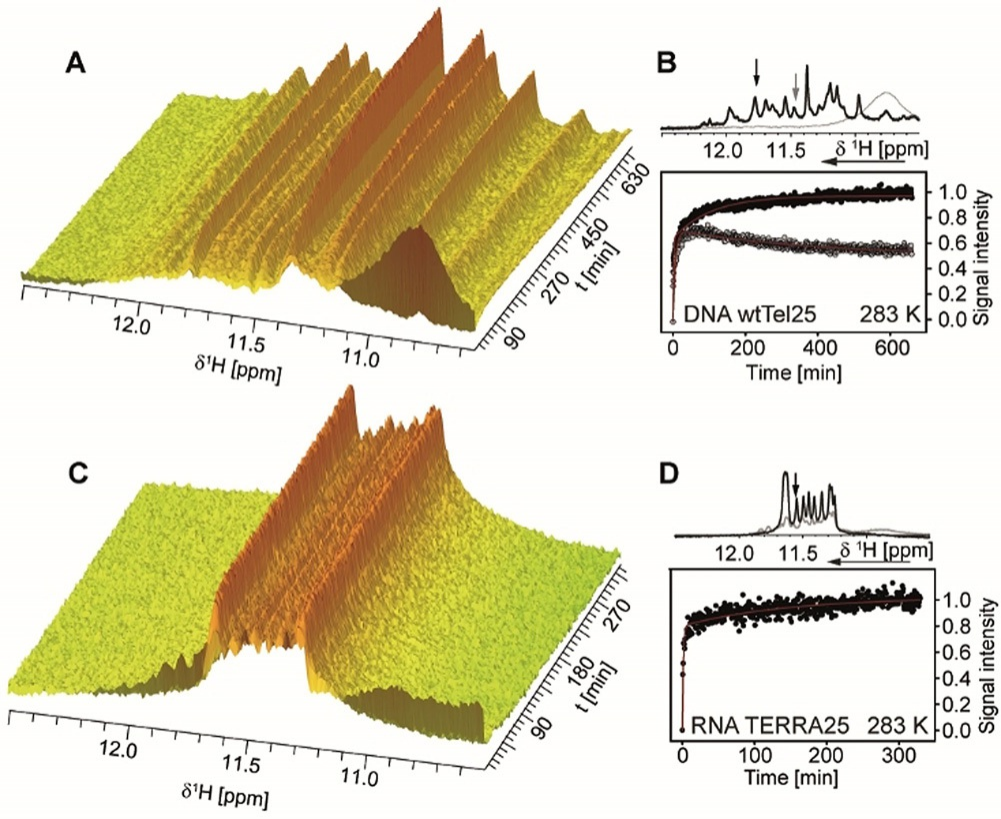
A) Change of the imino signal intensities over time of wtTel25 after KCl addition. B) Change of signal intensity over time for one selected signal for major (black) and minor (grey) conformation of wtTel25 indicated by arrows on top of the 1D ^1^H NMR spectra before (grey) and after (black) KCl addition. C) Change of the imino signal intensities over time of TERRA25 after KCl addition. D) Change of the signal intensity over time for a selected imino signal of TERRA25. Experimental conditions: 100 μM sample in 25 mM BisTris⋅HCl buffer (pH 7.0). Folding was induced by addition of KCl up to a final concentration of 15 mM. This low concentration can decrease G4‐oligomerisation. The spectra have been recorded at 283 K at 700 MHz with jump‐return water suppression.^[20]^ DSS was used as internal reference.

Here, we investigated folding of TERRA25 RNA G4 and the homologous wtTel25 DNA G4, as they have identical sequence but for the RNA(U) to DNA(T) substitution. wtTel25 undergoes biphasic folding kinetics resulting in the formation of a thermodynamically favoured major and a kinetically more rapidly formed, but less stable minor conformation with population ratios 0.6:0.4 at 283 K (Figure 1 A,B and Figure S2, Table S1), in line with previous studies for wtTel26.^[4]^ The Phan group has identified the major conformation of wtTel25 as hybrid‐2 (HT2, Figure 4 B). In analogy to our previous investigations, the minor population of wtTel25 most likely adopts a hybrid‐type structure but this has not been assigned so far.[^4^, ^19^]

For the RNA G4 TERRA25 (Figure 1 C,D and Figure S3, Table S1), we observed 3–4 times faster folding (*k*_1_=1.45±0.40 min^−1^ at 283 K) than for the DNA G4 wtTel25 (*k*_1_=0.41±0.32 min^−1^ at 283 K). The kinetic traces at *T*=283 K show a rapid kinetic phase and a slow signal equilibration towards the equilibrium population over several hours. By NMR, we observe a single folded state for TERRA RNA G4s. A second slow phase with amplitude change of ≈5 % is apparent in the kinetic trace (Figure 1 D). We attribute this slow second phase of low amplitude observed at high NMR concentrations to be caused by dimerization of TERRA25 at low temperature. In non‐denaturing polyacrylamide gel electrophoreses (PAGE) (Figure S4) and CD‐melting curves (Figure S14), we observed such dimerization tendencies for TERRA25 with a melting point of dimer around 18 °C. Thus, this second slow phase for TERRA G4 reports on dimerization that is not observed in the DNA wtTel25.

### Titration with KCl to wtTel25 and TERRA25

**Figure 2:**
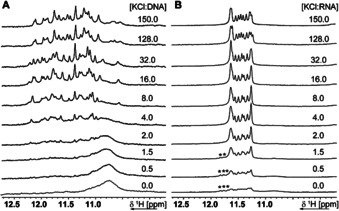
^1^H NMR titration of A) wtTel25 and B) TERRA25 with increasing amount of K^+^ recorded at 283 K and 600 MHz with jump‐return water suppression.^[20]^ A concentration of 100 μM DNA or RNA was used in 25 mM BisTris pH 7.0 and KCl was added stepwise. Equivalents correspond to strand concentration. 2 h for DNA G4 and 25 min for RNA G4 have been applied at room temperature for equilibration, wtTel25 and TERRA25 show differences in the K^+^‐free state and in the cooperativity upon addition of K^+^. Signals arising from another conformation than the parallel G4 of TERRA25 were marked with stars in B).

We performed ^1^H‐NMR titration of KCl to wtTel25 and TERRA25 to determine their K^+^‐binding cooperativity. Already the unfolded K^+^‐free states reveal differences for wtTel25 and TERRA25. In the absence of K^+^, only unspecific Hoogsten base pairs (broad signal between 10.5–11.2 ppm) can be detected for DNA wtTel25 while TERRA25 shows signals of the parallel folded G4 as well as signals stemming from another conformation visible in the region of 11.65–11.85 ppm even in the absence of K^+^ revealing partial prefolding of quadruplex‐like structure (indicated with stars in Figure 2). For TERRA25, unfolded conformations are converted into the folded state already upon addition of only 2.0 equiv KCl. In general, TERRA25 has a higher binding cooperativity towards K^+^ as wtTel25. Complete folding for TERRA25 was reached at 4 equiv KCl, and only at 32 equiv for wtTel25.

A similar behaviour was previously observed in CD‐titration on comparable human telomeric DNA and RNA sequences.^[21]^ We thus propose that TERRA25 rapidly undergoes hydrophobic collapse as no *syn*/*anti*‐rotation is required to adopt the stable parallel G4 structure.

Interestingly, further chemical shift perturbations (CSPs) can be observed by higher K+‐concentration for signals assigned to the lower and upper tetrads (around 11.3 ppm) (Figure S5–7). 2D data revealed the existence of a second and probably a third long‐lived conformation at lower K^+^‐concentration presumably due to differences in the capping structures (Figure S8,9).

### *χ* Angle Conformation of Guanosine Residues in the Unfolded States of G4

DNA G4 and RNA G4 adopt different quadruplex structures. RNA G4 exhibit parallel G4 strand orientations (Scheme 1 B). In this strand arrangement, all glycosidic torsion angles *χ* adopt an *anti* conformation. If the conformation of *χ* angles for guanosine residues in the K^+^‐free unfolded state of G4 RNAs was also *anti*, then slow *anti*/*syn* conformational transitions do not have to take place during the K^+^‐induced folding of G4 RNAs.

**Figure 3:**
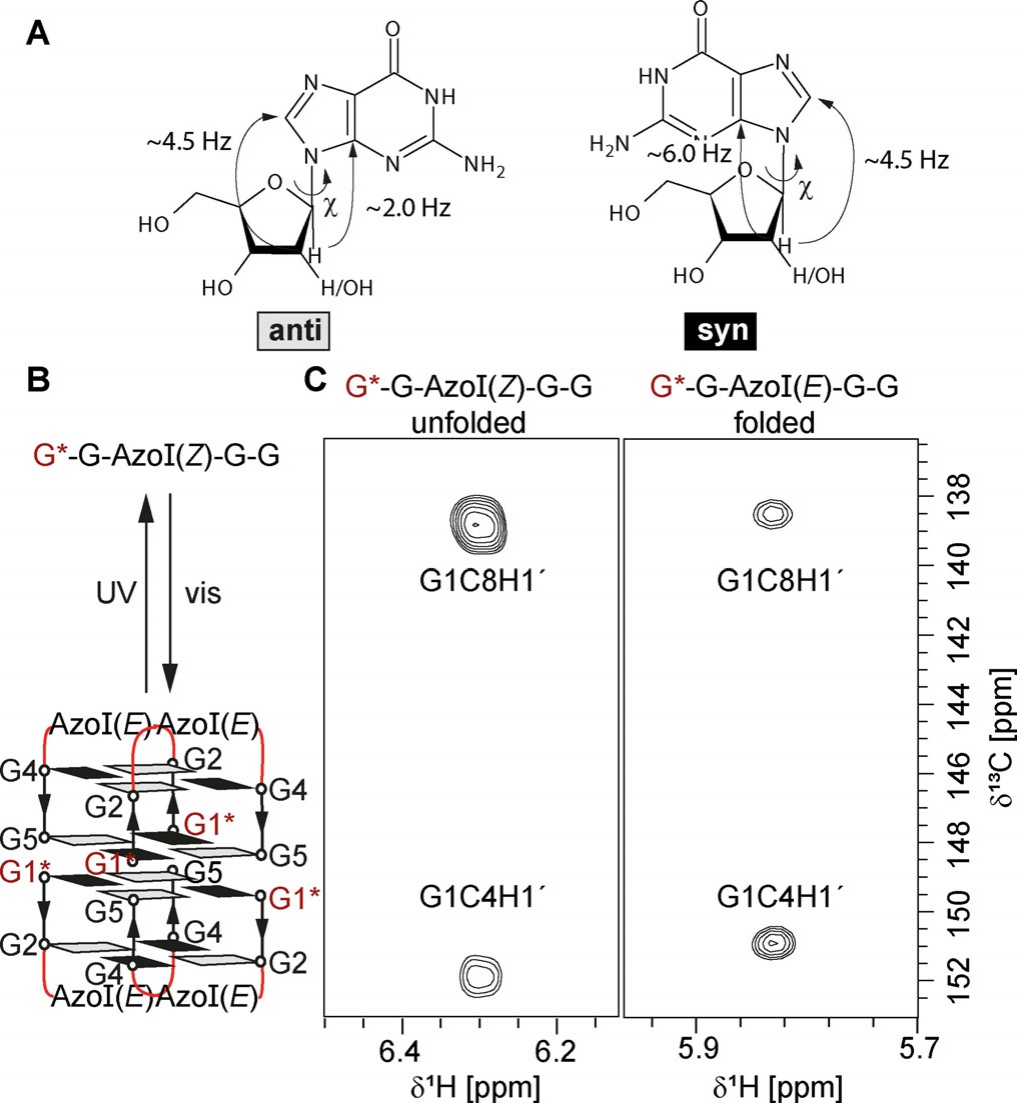
A) *Anti* and *syn* conformation of the glycosidic torsion angle *χ* of guanosine and 2′deoxyguanosine with corresponding ^3^ *J*(C4,H1′)‐ and ^3^ *J*(C8,H1′)‐ coupling constants. B) Photoswitchable DNA G‐quadruplex (AzoG4). The stable *E*‐configuration of the N=N‐azo bridge (AzoI) forms an antiparallel tetramolecular G4 with four tetrads that switches into the unfolded *Z*‐configuration upon UV‐irradiation.^[25]^ G1* is ^13^C,^15^N‐labelled C) Regions of long‐range ^13^C‐HMQC‐spectra showing the G1C8H1′ and G1C4H1′ cross peak region of unfolded AzoG4 and folded AzoG4. Residue G1 adopts a *syn* conformation (pdb‐code: 2N9Q) in the G4 DNA which results in a strong C4H1′ cross peak and a weak C8H1′ cross peak. The reverse is observed in the unfolded conformation. Experimental conditions: 260 μM AzoG4 in 25 mM dTris⋅HCl (pH 7) containing 15 mM KCl in 100 % D_2_O, DSS was used as reference substance.

Very early on, conformational preferences of phosphorylated guanosines and 2′‐deoxyguanosines in mononucleotides and in unfolded states of RNA and DNA have been investigated in theoretical studies by Olsen^[22]^ proposing the presence of both *syn* and *anti* conformations, but systematic experimental investigations are missing. Thus, we here determined vicinal ^3^ *J*(C8,H1′)‐ and ^3^ *J*(C4,H1′)‐coupling constants that depend on *χ* (Figure 3 A). ^3^ *J*(C8,H1′) is 4.5 Hz for both, *syn* and *anti* conformation, but ^3^ *J*(C4,H1′) in *syn* conformation is 6.0 Hz, and thus larger than the ^3^ *J*(C4,H1′) of 2.0 Hz reported for *anti* conformation.^[23]^ Qualitatively, if the C8H1′ coupling is larger than the C4H1′ coupling, the nucleotide adopts (predominantly) an *anti* conformation while if the ^3^ *J*(H1′,C8) coupling is smaller than ^3^ *J*(H1′,C4) coupling, it adopts (predominantly) a *syn*‐conformation (Figure 3 A). For the mononucleotides rGMP^[24]^ and dGMP (Figure S10,11, Table S2), the *anti* conformation is the predominant conformation. We further measured these ^3^ *J*(C,H) couplings for a DNA G4 with a single ^13^C,^15^N‐labelled guanosine whose conformation can be triggered by light between the folded G4 DNA conformation and an extended unfolded conformation in presence of KCl. The photoswitching behaviour of this model G4 DNA relies on the configuration of an azo‐group that switches between the stable (*E*) conformation that enables G4 folding and the (*Z*) conformation upon UV irradiation that unfolds G4.^[25]^ The G4 structure has been determined by NMR (pdb‐code: 2N9Q) and it is known that the ^13^C,^15^N‐labelled G1 (red color code in Figure 3 B) adopts *syn*‐conformation in the folded state.^[25]^ The observed significant change of the signal intensities in the long‐range HMBC that report on ^3^ *J*(C4,H1′) and ^3^ *J*(C8,H1′) shows that not only nucleotide monophosphates have an *anti*‐preference but also nucleotides in unfolded G4 (Figure 3 C). Since both dGMP and rGMP have a preference for the *anti*‐glycosidic conformation, we assume that RNA nucleotides in unfolded G4 adopt *anti* conformation as well.

Further, we performed a temperature jump experiment on wtTel25. These experiments reveal no differences between low K^+^ and high K^+^ conditions (Figure S12,13, Table S3). These observations provide a basis for discussing our biophysical findings to conditions where folding was induced at constant K^+^ concentrations.

### Structure Modulation of DNA G4 by Introduction of 2′F‐ANA Guanosine Residues

**Figure 4:**
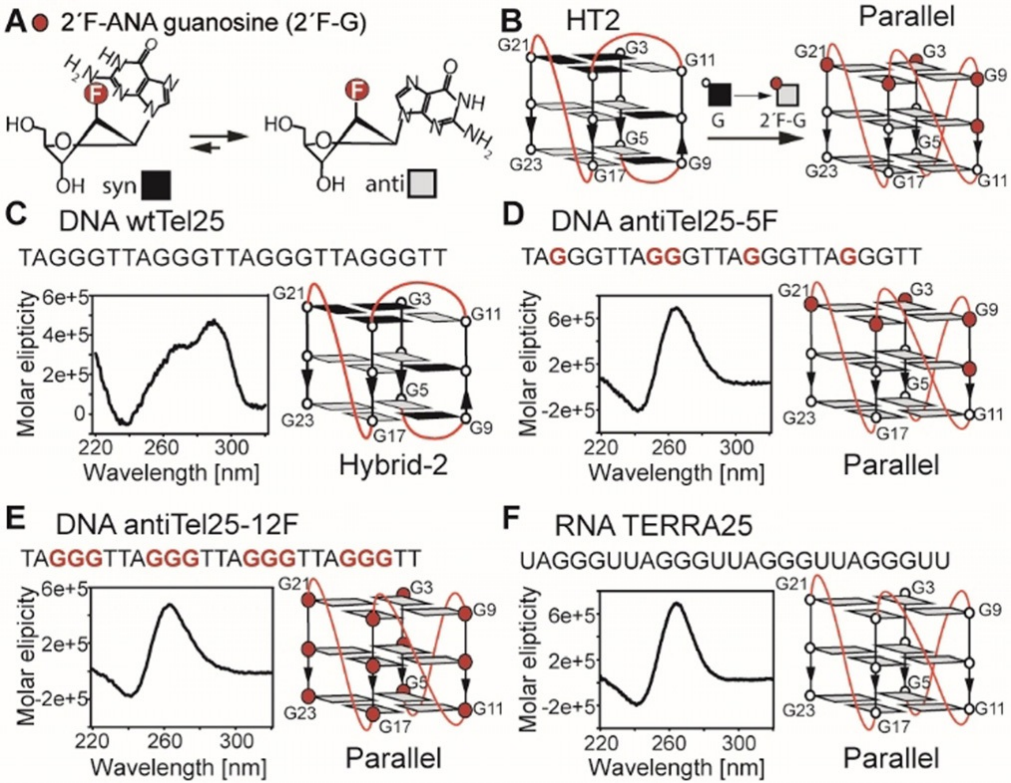
A) Chemical constitution of 2′F‐arabino (ANA) guanosine (displayed as red filled circles) in southeast sugar pucker conformation with preference of the *anti* conformation. Guanosine in *syn* conformation are displayed as black filled rectangles and in *anti* conformation as grey filled rectangles. B) Left: the hybrid‐2 (HT2) conformation adopted by wtTel25; right: the proposed parallel conformation of antiTel25‐5F that results from the replacement of the 5 guanosines in *syn* conformation in wtTel25 by 2′F ANA guanosines. C) Sequence and CD spectra of wtTel25, D) antiTel25‐5F, E) antiTel25‐12F and F) TERRA25. The adopted major conformation is shown on the right of each spectrum. Experimental conditions: 10 μM sample, 25 mM potassium phosphate buffer pH 7.0, the sample was prepared a day prior to the measurement.

The comparison of DNA and RNA G4 folding kinetics reveals faster folding kinetics and no intermediates on the folding pathway of RNA G4. 2′‐deoxy‐2′F‐arabino‐ modified guanosines (2′F‐ANA) forces the nucleobase into *anti* conformation (Figure 4 A).^[26]^ By studying structure and folding kinetics of this DNA derivatives, we can force the DNA sequence into adopting a parallel‐type G4 with the same sequence as wtTel25 (Figure 4 B).^[27]^ 2′F‐ANA G adopts a southeast conformation of the sugar pucker^[28]^ and an *anti*‐glycosidic torsion angle to reduce the steric clash between the fluorine atom and the nucleobase.^[29]^

In a first construct (antiTel25‐5F), we substituted those five 2′‐deoxyguanosines that adopt *syn* conformation in the HT2 structure of wtTel25 (G3, G9, G10, G11 and G21) by 2′F‐ANA guanosine (Figure 4 B and D) following previous reports that substitution of guanosines in *syn* conformation only is sufficient to change the overall structure.[^27^, ^30^] In a second construct, we substituted all 2′‐deoxyguanosines by 2′F‐ANA guanosines (antiTel25‐12F) (Figure 4 E). The previously reported structural change was confirmed by CD spectroscopy (Figure 4 C–F) as G4s with hybrid and parallel strand orientation show substantially different CD profiles. Both modified constructs show the CD‐signal characteristics for the parallel conformation with a maximum at 265 nm and a minimum at 245 nm, different to hybrid conformations with maxima at 290 nm and 265 nm and a minimum around 240 nm.^[31]^

### Thermal Stability

The melting temperatures (*T*_m_) for the four G4 constructs range between 48 °C for wtTel25 and 91 °C for antiTel25‐12F. Even though two conformations have been determined for wtTel25, only a single transition can be observed in CD melting (Figure S14), arguing for a small delta *T*_m_ between minor and major conformation, which is further supported by the near 1:1 population of both conformations. AntiTel25‐5F has a stabilised major conformation with a melting temperature at 61 °C. CD melting curves showed two transitions at 18 °C and at 64 °C for TERRA25 (Figure S14) as well as two transitions for antiTel25‐12F at 43 °C and at 91 °C (Figure S14). The high thermal stability of antiTel25‐12F is not surprising. It has been previously observed that the introduction of 2′F ANA guanosine has a stabilising effect on the G4 structure by around 1 °C per modification but also a thermal stabilisation of 12 °C for a single G to 2′F‐ANA G modification was observed.[^30^, ^32^] The additional stabilisation is determined by an F‐H8 pseudo‐hydrogen‐bond and F‐CH‐O4′ electrostatic interaction.^[32]^

### Kinetics of antiTel25‐5F and antiTel25‐12F

**Figure 5:**
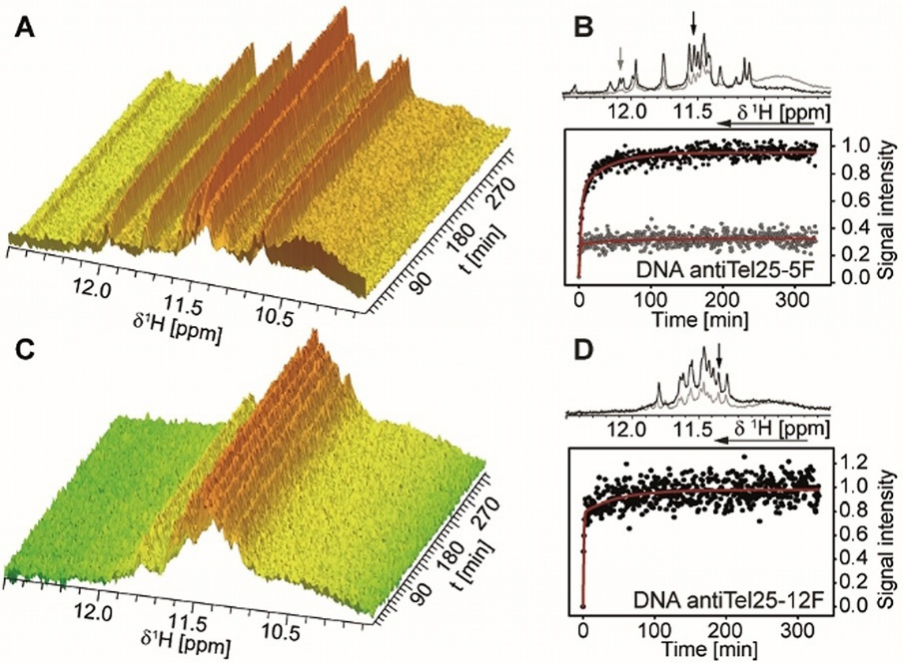
A) Change of the imino signals of antiTel25‐5F over time upon KCl addition. B) Change of the signal intensities over time for selected signal of the major conformation (black) and the minor conformation (grey) of antiTel25‐5F. The signals are indicated with arrows on top of the 1D ^1^H NMR spectrum before (grey) and after (black) KCl addition. The minor conformation of antiTel25‐5F is fully converted into the major conformation after at least 1.5 day at room temperature (Figure S17) C) Change of the imino signal intensities over time of antiTel25‐12F after KCl addition. D) Change of the signal intensity over time for a selected imino signal of antiTel25‐12F after KCl addition.

We recorded the K^+^‐induced folding kinetics of antiTel25‐5F and antiTel25‐12F at 283 K (Figure 5 and Figure S15,16, Table S1). While the kinetics of antiTel25‐5F folding is similar to DNA G4 wtTel25, folding kinetics of antiTel25‐12F resembles the kinetics of RNA G4 TERRA25. For antiTel25‐5F we observe kinetic partitioning of the folding and the rate constants of both conformations (*k*_1_=0.38±0.09 min^−1^ and 0.68±0.16 min^−1^ at 283 K for major and minor (0.75:0.25) conformation, respectively) are not significantly increased compared to the kinetics of DNA G4 wtTel25. The minor conformation of antiTel25‐5F is less stable and has been fully converted into the major conformation (Figure S17). By contrast, in antiTel25‐12F the formation of any intermediate with G in *syn* conformation is completely supressed and fast folding into one distinct conformation with a comparable folding rate (*k*_1_=1.69±0.43 min^−1^ at 283 K) to the folding rate of RNA G4 TERRA25 was indeed observed. The folding kinetics of antiTel25‐12F show biphasic behavior. CD melting curve (Figure S14) suggest that antiTel25‐12F has similar dimerization tendency as TERRA25. In comparison, the stabilised parallel c‐*MYC22* (PDB: 1XAV) DNA G4 has a fast folding rate but the kinetic of its folding still reveal kinetic partitioning and a comparison of tetrameric DNA and RNA G4 showed faster assembling of RNA G4.^[33]^ Altogether, the data support the hypothesis of a complete lack of any guanosine with *syn*‐glycosidic conformation on the TERRA G4 folding landscape.

### Reaction Coordinates

**Figure 6:**
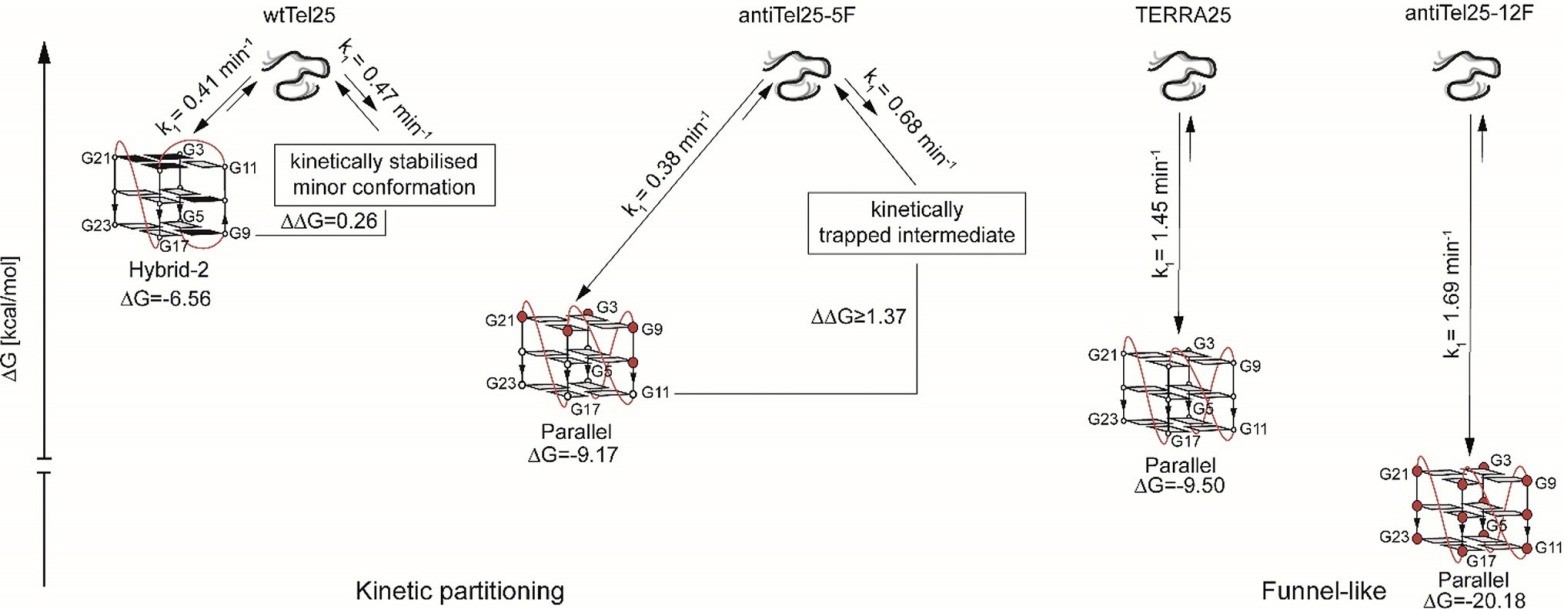
Reaction coordinate of folding landscape of DNA, RNA and modified G4s. The values are based on folding experiments, van't Hoff analysis of melting experiments and population ratios as discussed in the main text. The structures of the minor conformation of wtTel25 and antiTel25‐5F have not been determined. The kinetic measurements have been performed at 15 mM K^+^‐concentration and the thermodynamic parameters have been determined in 40 mM K^+^‐concentration. The absolute values may differ with the K^+^‐concentration but stay comparable within the constructs.

Beside the melting point, a van't Hoff analysis of the melting curves was performed to determine Δ*H*°, Δ*S*° and Δ*G*° (Table S4). As TERRA25, antiTel‐25‐5F and antiTel25‐12F exhibit a high melting point, the baseline might be insufficient for an accurate determination of Δ*H*°, Δ*S*° and Δ*G*° and this approach has its limitation when more than two states are involved. So, the data have to be handled with care but give an estimation of the relative free energy of the constructs. Furthermore, the population of the conformations has been determined by integration of well resolved signals in ^1^H‐NMR spectra (Figure S18) to calculate ΔΔ*G* of the involved conformations. Together with the folding rates, this complete set of thermodynamic and kinetic data enables us to propose reaction coordinates for the folding landscape of the different G4 constructs (Figure 6). The DNA G4 wtTel25 folds into the thermodynamically stable hybrid‐2 and a second kinetically stabilised hybrid structure which have similar free enthalpies (ΔΔ*G*=0.26 kcal mol^−1^). The antiTel25‐5F construct folds into a parallel G4 and forms a kinetically trapped intermediate with a final population ratio major/minor of at least 0.92:0.08 (ΔΔ*G*≥1.37 kcal mol^−1^) as derived from the detection limit due to S/N in the ^1^H‐NMR spectra (Figure S18). wtTel25 and antiTel25‐5F follow a kinetic partitioning folding mechanism, while the folding landscapes of antiTel25‐12F and TERRA25 are funnel‐like with a parallel G4 as final structure. The parallel structure of antiTel25‐12F is significantly more stable than the one of TERRA25 due to stabilising‐effect of 2′F‐ANA guanosine discussed above.^[30]^

## Conclusion

Human telomeric RNA G4s have a fundamentally different folding landscape than human telomeric DNA G4s. Five a*nti*‐directing modifications were introduced in a 25mer DNA G4‐forming sequence at positions with guanosines in *syn* conformation allowing us to drive the DNA structure from a hybrid towards a parallel G4 conformation as observed for RNA G4s. However, replacing five deoxyguanosine residues was not sufficient to completely reshape the folding landscape from the typical DNA G4 folding landscape with kinetic partitioning to the RNA G4 funnel‐like folding landscape. Only if all 12 deoxyguanosine residues are replaced by 2′F‐ANA guanosines the DNA G4 folding landscape changes from a kinetic partitioning to a funnel‐like folding landscape. The experimental data thus support our hypothesis that RNA G4s have a higher propensity for the *anti*‐glycosidic conformation both in the unfolded and the folded state and no other than the parallel G4 structure is formed, even not transiently during folding. On the contrary, DNA G4 have a thermodynamic preference for the *syn*‐glycosidic conformation. Only if the *syn*/*anti* rotation is prevented by constraining all the G‐quartet forming nucleotides in *anti* conformation, the DNA G4 folding behaviour is comparable to the one of RNA G4. Recently, it has been shown that the folding rate is also influenced by the loop length.^[34]^ Hence, both factors the required syn/anti flipping and the loop length have to be taken into account to estimate G4 folding rates and the potential population of long‐lived intermediates on the folding landscape.

## Conflict of interest

The authors declare no conflict of interest.

## Supporting information

As a service to our authors and readers, this journal provides supporting information supplied by the authors. Such materials are peer reviewed and may be re‐organized for online delivery, but are not copy‐edited or typeset. Technical support issues arising from supporting information (other than missing files) should be addressed to the authors.

Supplementary
