# Supplementary material for: The Folding Landscapes of Human Telomeric RNA and DNA G‐Quadruplexes are Markedly Different
Source: Angew Chem Int Ed Engl. 2021 Apr 6;60(19):10895–901. doi: 10.1002/anie.202100280 (PMC8252441; doi:10.1002/anie.202100280)
Supplement: Supplementary file 1 — Supplementary [file ANIE-60-10895-s001.pdf]

Angewandte Chemie
Eine Zeitschrift der Gesellschaft Deutscher Chemiker

Supporting Information

The Folding Landscapes of Human Telomeric RNA and DNA G-Quadruplexes are Markedly Different

*Diana Müller, Irene Bessi, Christian Richter, and Harald Schwalbe**

anie_202100280_sm_miscellaneous_information.pdf

Table of Contents

Experimental Procedures

Sample preparation

Native polyacrylamide gel electrophoreses

KCl-induced NMR folding experiments

NMR folding experiments with temperature jump

^{1}H NMR titration

2D NMR experiments for assignment of TERRA25

2D NMR experiments to analyse the influence of KCl-concentration on the structure of TERRA25

NMR experiments to characterise the glycosidic conformation in the unfolded state

Circular dichroism (CD) experiments

Results and Discussion

Kinetic traces and rate constants determined by KCl-induced folding

Figure S1: wtTel25 and TERRA25 at 298 K, 288 K and 283 K

Figure S2: Kinetic traces of wtTel25 at 283 K

Figure S3: Kinetic traces of TERRA25 at 283 K

Table S1: rate constants for wtTel25, TERRA25, antiTel25-5F and anti Tel25-12F

Native polyacrylamid gel

Figure S4: Native gel of TERRA10, TERRA12 and TERRA25

Attempt of assignment of TERRA25

Figure S5: ^{1}H,^{13}C-HSQC

Figure S6: ^{1}H,^{1}H-NOESY

Figure S7: D_2O-exchange

2D NMR data to analyse effect of KCl on the structure of TERRA25

Figure S8: Overlaid ^{1}H,^{1}H-NOESY of TERRA25 recorded at 4 equiv and 128 equiv KCl

Figure S9: ^{13}C-HSQC recorded at 4 equiv KCl

Analysis of the glycosidic conformation of dGMP

Figure S10: ^{1}H,^{13}C-HMBC of rGMP and dGMP

Figure S11: ^{1}H,^{13}C-HMQCs of dGMP recorded with different delays

Table S2: Calculated ^3J(H1´,C4) and ^3J(H1´,C8)

Analysis of folding rates of wtTel25 determined by temperature jump-induced folding

Figure S12: 1D ^{1}H spectrum of the aromatic region of wtTel25 at 318 K and 308 K.

Figure S13: Kinetic traces of wtTel25 recorded at 308 K after temperature jump from 318 K

Table S3: rate constants for wtTel25 for temperature jump induced folding

CD melting curves

Figure S14: CD melting and annealing curves

Kinetic traces of modified DNAs determined by KCl-induced folding

Figure S15: Kinetic traces of antiTel25-5F at 283 K

Figure S16: Kinetic traces of antiTel25-12F at 283 K

Conversion of antiTel25s minor conformation

Figure S17: 1D ^{1}H NMR spectra of antiTel25-5F after kinetic measurement and 1.5 days later

Thermodynamic parameters

Table S4: T_m, ΔH°, ΔS°, ΔG° of wtTel25, antiTel25-5F, TERRA25 and antiTel25-12F and ΔΔG of wtTel25 and antiTel25-5F

Population of minor and minor conformation of wtTel25 and antiTel25-5F

Figure S18: 1D ^{1}H NMR spectra of wtTel25 and antiTel25-5F

References

Experimental Procedures

Sample preparation

The DNA wtTel25 (TAGGGTTAGGGTTAGGGTTAGGGTT) is purchased from Eurofins MWG Operon (Ebersberg, Germany) in HPSF (high purity salt free) grade. The 2´deoxy-2´F-arabinoguanosine (2´F-ANA G or F) modified DNAs antiTel25-5F (TAFGGTTAFFGTTAFGGTTAFGGTT) and antiTel25-12F (TAFGGTTAFFGTTAFGGTTAFGGTT) were purchased from IBA-lifesiences (Göttingen Germany) and the RNA TERRA25 (UAGGGUUAGGGUUAGGGUUAGGGUU) from Dharmacon (Cambridge, UK). The oligomers have been HPLC purified with tetrabutylammonium acetate buffer, desalted with ultracentrifuge filtration devices (Vivacon (2 kD cut-off), VWR) and precipitated with 5 volumes of $LiClO_4$ (2% in Acetone (w/v)) at -20 °C over night. Subsequently, another desalting step has been performed to remove residual Li^+ and acetone.

Native polyacrylamide gel electrophoreses (PAGE)

The folded state of TERRA25 was further characterised with a native PAGE (15% acrylamide gel). For analysis ~ 100 pmol were loaded in 40% glycerol and 50 mM Tris·Acetate pH 8.3 with 5 mM KCl. Gels were prepared in 50 mM Tis·Acetate buffer pH 8.3 containing 5 mM KCl. Bands were separated in the same buffer at constant power (< 1 W) for 4 h with water cooling (13 °C water temperature). The bands have been visualised with StainsAll (SigmaAldrich).

KCl-induced NMR folding experiments

For the mixing experiments, the DNA or RNA was provided as 100 µM solution in 300 µL of 25 mM BisTris·HCl buffer pH 7.0. The variation of imino peak intensity was monitored as function of the time as pseudo-2D experiment with jump-return water suppression[1] recorded at 700 MHz (Bruker) equipped with a prodigy cryogenic probe. During the measurement, a KCl solution with 15 mM final concentration was added using a rapid mixing device. 1D ^1H NMR spectra with jump return water suppression[1] were recorded with a time resolution of ~ 1.1 sec/scan. In order to improve the S/N ration, the spectra have been added up to 4 (wtTel25 at 298), 8 (TERRA25 at 298 K) or 32 scans. All NMR data were collected, processed and analysed using the software TopSpin 3.6.2 (Bruker), R x64 3.6.3 and Sigma Plot 12.5. Thereby, a script was used performing a baseline correction of every 1D spectrum individually. The kinetic traces were normalized and optimal fitted with biexponential fit (equation 1).

$$(1) \qquad y(x) = a(1 - e^{-bx}) + c(1 - e^{-dx})$$

NMR folding experiments with temperature jump

For the temperature jump experiments, the DNA was provided as 100 µM solution in 300 µL of 25 mM BisTris·HCl buffer pH 7.0 containing 15 mM KCl. Nearly complete unfolding of wtTel25 was achieved at 318 K and a temperature jump to 308 K was performed at 700 MHz (Bruker) equipped with a prodigy cryogenic probe. 80 seconds have been applied for temperature equilibration then the variation of aromatic peak intensity was monitored as function of the time as pseudo-2D experiment with excitation sculpting water suppression[2] recorded at 700 MHz (Bruker). 4 Scans per 1D with a time resolution of ~ 4.5 sec. In total 6 repetition have been performed and summed up in order to improve the S/N ratio. All NMR data were collected, processed and analysed using the software TopSpin 3.6.2 (Bruker), R x64 3.6.3 and Sigma Plot 12.5. Thereby, a script was used performing a

baseline correction of every 1D spectrum individually. The kinetic traces were normalized and optimal fitted biexponential (equation 2) for decreasing unfolded signals or monoexponential (equation 3) for increasing signals.

(2) $$y(x) = ae^{-bx} + ce^{-dx}$$

(3) $$y(x) = a(1 - e^{-bx})$$

^{1}H NMR titration

100 µM wtTel25 or TERRA25 have been provided in 25 mM BisTris·HCl pH 7.0 in 90% H_2O/10% D_2O. ^{1}H NMR spectra have been recorded with jump-return water suppression[1] at 600 MHz with 128 scans per spectrum. 25 min for TERRA25 and 2 h for wtTel25 have been applied at room temperature for equilibration after each KCl-addition. All NMR samples were referenced with 2,2-dimethyl-2-silapentane-5-sulfonate (DSS).

2D NMR experiments for assignment of TERRA25

2D ^{1}H,^{13}C-HSQC and 2D ^{1}H,^{1}H-NOESY spectra with 200 ms mixing time were recorded on a 200 µM sample either in 90% H_2O/10% D_2O or 100% D_2O and 25 mM BisTris·HCl pH 7.0 buffer containing 15 mM KCl at 298 K and 600 MHz. Excitation sculpting was used for water suppression.[2] The data were analysed using Sparky.[3]

2D NMR experiments to analyse the influence of KCl-concentration on the structure of TERRA25

Two ^{1}H,^{1}H-NOESY spectra have been measured at 283 K with 4k points in direct and 1k points in indirect dimension and 40 scans. Mixing delay was 200 ms and excitation sculpting was used for water suppression.[2] A 200 µM sample in 90% H_2O/10% D_2O and 25 mM BisTris·HCl pH 7.0 buffer was used containing either 4 equiv KCl or 128 equiv KCl. The data were analysed using Sparky.[3] A ^{13}C-HSQC was recorded at 4 equiv KCl.

NMR experiments to characterise the glycosidic conformation in the unfolded state

For the determination of long-range proton-carbon couplings, a sample of 10 mM 5´-dGMP in 25 mM BisTris buffer (pH 7.0) containing 15 mM KCl have been prepared. A refocused HMQC[4,5] with magnetization transfer delays of 41 ms, 58.2 ms and 59 ms was recorded for 5´-dGMP. ^{3}J(H1´C4) and ^{3}J(H1´C8) have been calculated based on equation 4 or 5, respectively with ^{1}J(H1´C1´) = 165.9 Hz after extraction of the signal intensities with TopSpin 3.6.2 (Bruker).

(4) $$^3J(H1', C4) = \frac{1}{\pi\Delta} * \arctan[\sqrt{\frac{I(H1', C4)}{I(H1', C1')}} * \tan(\pi ^1J(H1', C1')\Delta)]$$

(5) $$^3J(H1', C8) = \frac{1}{\pi\Delta} * \arctan[\sqrt{\frac{I(H1', C8)}{I(H1', C1')}} * \tan(\pi ^1J(H1', C1')\Delta)]$$

The photoswitchable DNA G4 (GG-Azo1-GG) has been synthesized in the Heckel group (Goethe University Frankfurt, Germany). G1 is ^{13}C,^{15}N-labelled. A 260 µM sample in 25 mM dTris*HCl (pH 7.0) and 15 mM KCl was used. Refocused ^{1}H,^{13}C-HMQC[4,5] with magnetization transfer delays of 60 ms were recorded before and after 5 min irradiation with UV-light (365 nm) at 600 MHz and 298 K.

Circular dichroism (CD) experiments

CD measurements have been performed using a JASCO J-810 spectropolarimeter equipped with a Peltier temperature control system. 10 µM DNA or RNA have been provided in 25 mM Potassium phosphate buffer pH 7.0.CD spectra between 220 and 320 nm have been recorded with a scanning speed of 100 nm/min and 10 accumulation. A quartz cuvette with 1 mm pathlength has been used and a baseline correction was performed. For melting and folding curves, a heating rate of 0.5 °C/min was used recorded at 290 nm for the hybrid G4 and 264.5 nm for the parallel G4. Sigmoidal fits were performed with SigmaPlot 12.5. Van´t Hoff analysis (equation 6) was used to determine ΔH°, ΔS° and ΔG° (equation 7):

(6) $$\ln(K_{unfolding}) = -\frac{\Delta H°}{RT} + \frac{\Delta S°}{R}$$

R = gas constant (8.314 J* K^{-1} * mol^{-1})

(7) $$\Delta G = \Delta H - T\Delta S$$

Results and Discussion

Kinetic traces and rate constants determined by KCl-induced folding

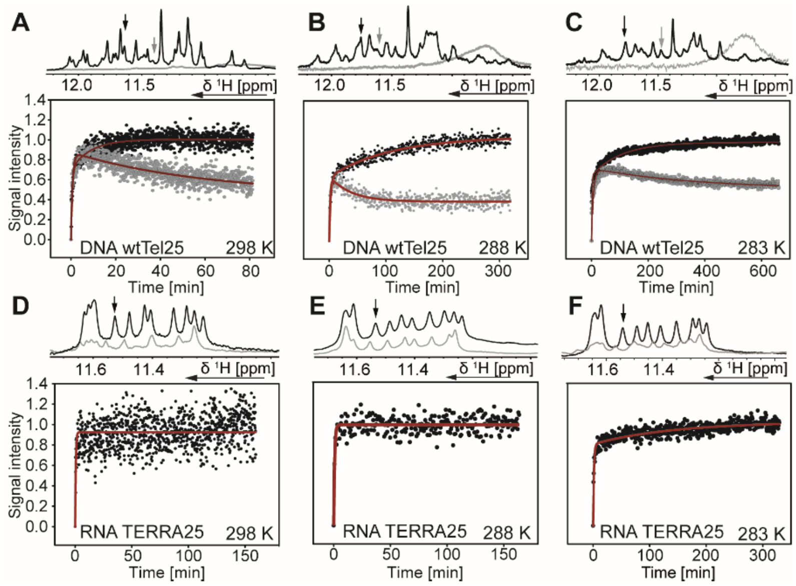

Figure S1: Kinetic traces of K$^+$-induced folding of DNA G4 wtTel25 at a) 298 K, b) 288 K and c) 283 K and of RNA G4 TERRA25 at d) 298 K, e) 288 K and f) 283 K. ^{1}H NMR imino region before (grey) and after (black) folding are displayed on top of each kinetic traces. The imino signal chosen for the kinetic analysis is marked with an arrow (black arrow: major conformation, grey arrow: minor conformation).

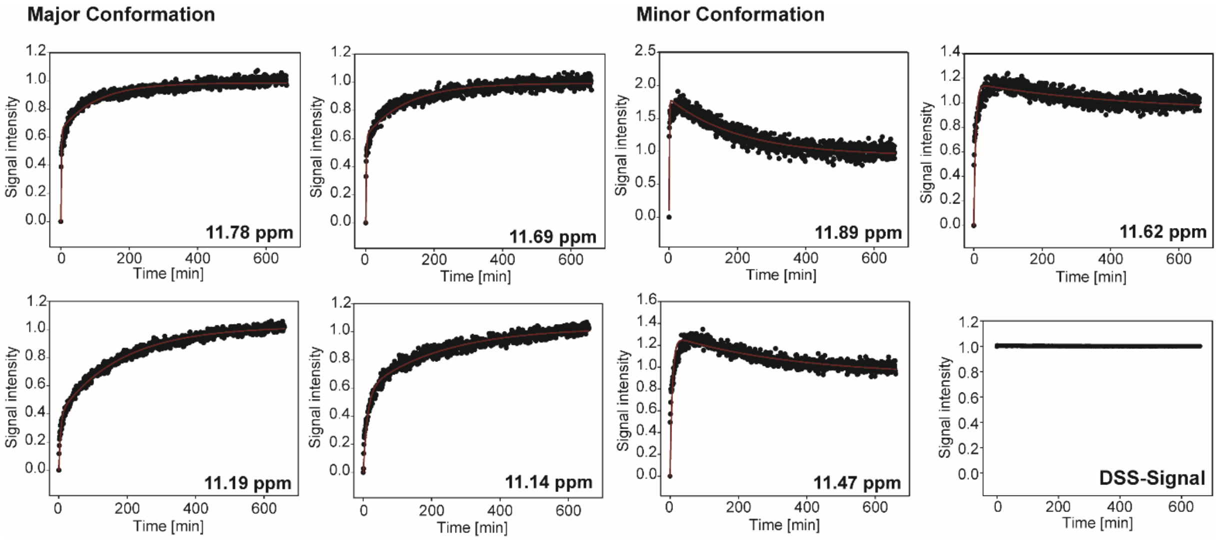

Figure S2: Kinetic traces that have been used for analysis of wtTel25 folding kinetics recorded at 283 K and the kinetic trace of the DSS signal that has been used as correction to eliminate any changes in spectra due to the mixing procedure.

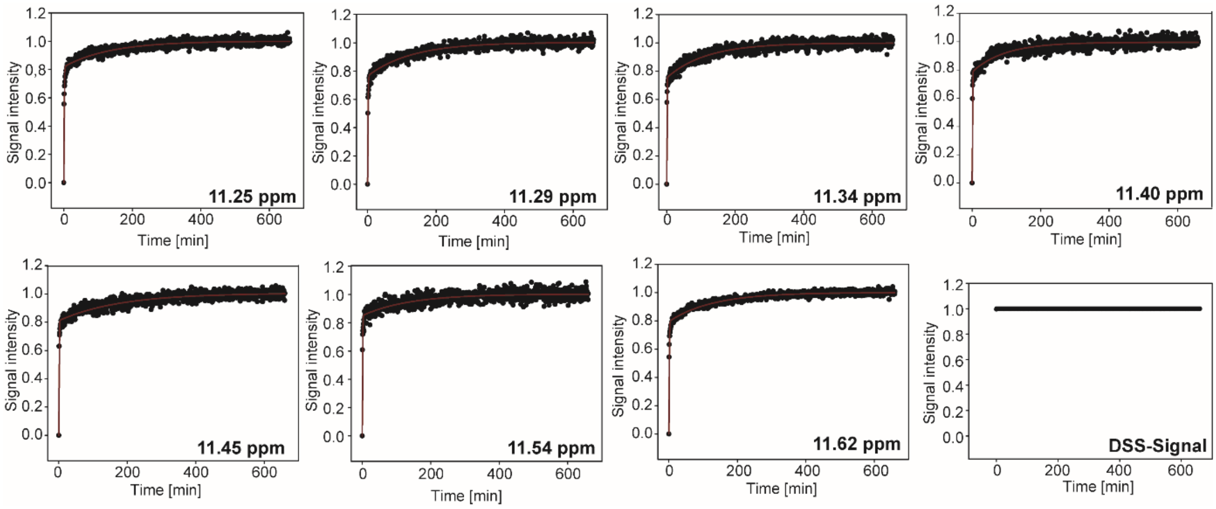

Figure S3: Kinetic traces of TERRA25 recorded at 283 K that have been used to determine the observed folding rate. The DSS signal intensity changes have been used as correction factor.

Table S1: mean values and standard deviation of the kinetic traces displayed in Figure S2-3, Figure S15 and S16.

	k [min^{-1}]	283 K
**wtTel25**	k_1	0.41± 0.32
	k_2	0.007 ± 0.003
Minor conformation	k_1	0.47 ± 0.52
	k_2	0.003 ± 0.002
**antiTel25-5F**	k_1	0.38 ± 0.09
	k_2	0.02 ± 0.01
Minor conformation	k_1	0.68 ± 0.16
	k_2	0.04 ± 0.03
**TERRA25**	k_1	1.45 ± 0.40
	k_2	0.008 ± 0.002
**antTel25-12F**	k_1	1.69 ± 0.43
	k_2	0.02 ± 0.01

Native polyacrylamid gel

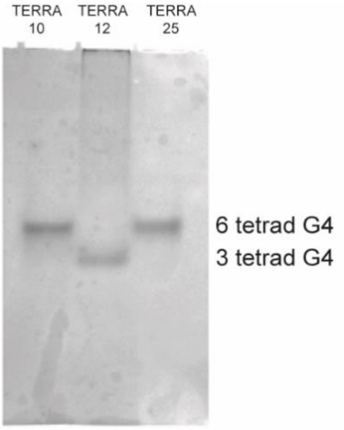

Figure S4: Native polyacrylamid gelelectrophoreses at 13 °C with samples of TERRA10 and TERRA12 as references and TERRA25. TERRA10 forms a tetramolecular six-tetrad G4 (PDB:2M18) and TERRA12 forms a bimolecular three-tetrad G4 (PDB:2KBP).[6] TERRA25 runs at the hight of the six-tetrad 10mer proving the dimeric nature of TERRA25.

Attempt of assignment of TERRA25

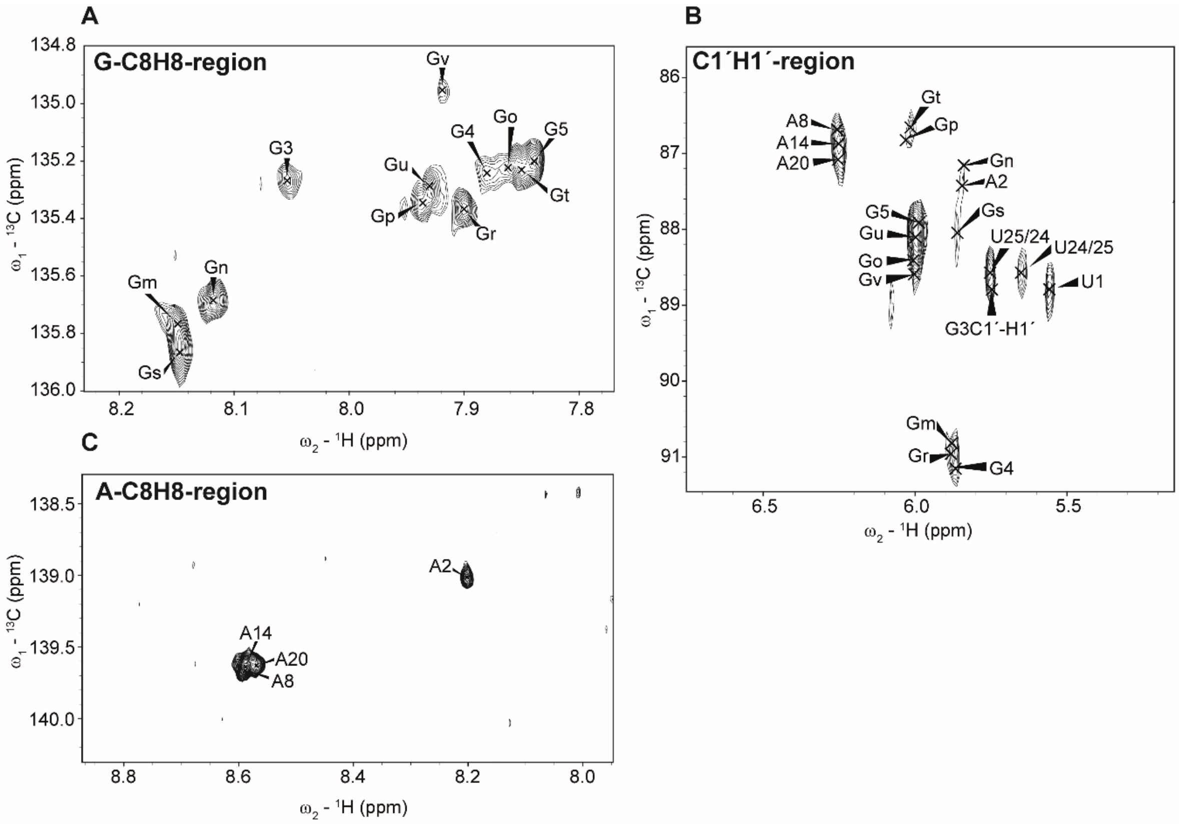

Figure S5: Extracts of ^{1}H,^{13}C-HSQC of TERRA25 of A) the aromatic region with average ^{13}C chemical shifts of guanosine C8, B) anomeric region and C) aromatic region of Adenosine C8H8.

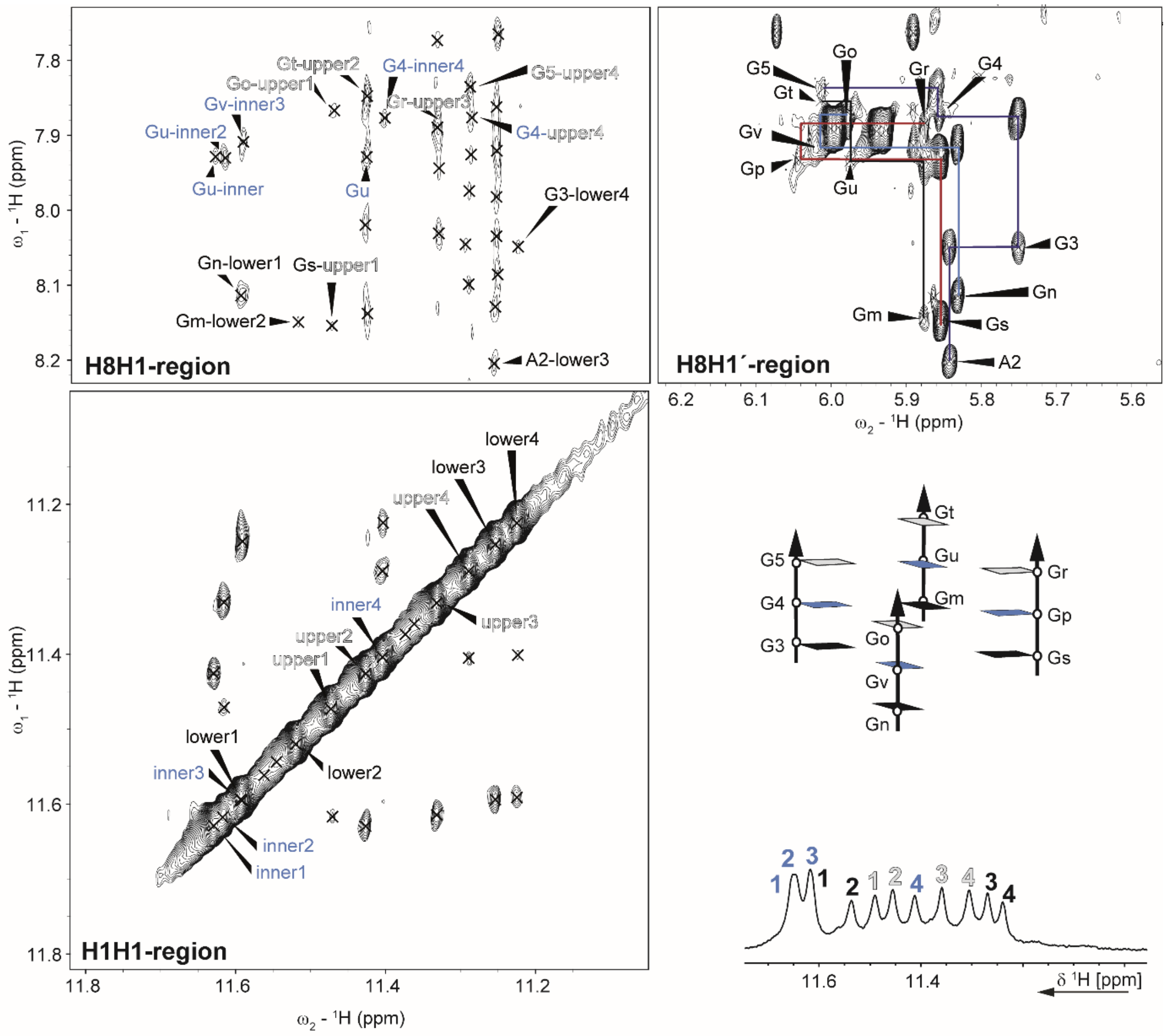

Figure S6: Extracts of $^{1}H,^{1}H$-NOESY spectrum of TERRA25.Upper right: The H1´H8-walk was used to assign the connected Gs (lower right). As the structure is nearly symmetric, all loop-Uridines are resonating with the same chemical shift impeding a full sequential walk. Upper left: H8-H1 cross signals, the strongest cross-signals are appearing within a tetrad allowing an assignment of the imino signals to the three tetrads (lower (black), inner (blue) and upper (grey) tetrad) (lower left and lower right).

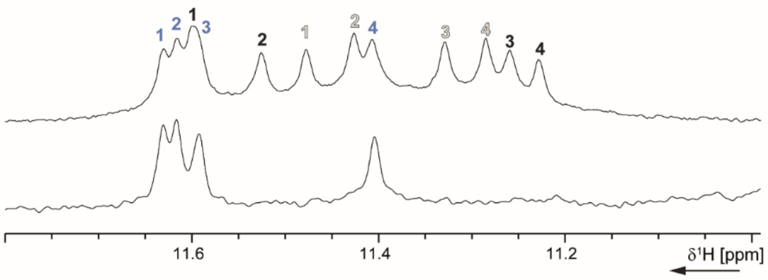

Figure S7: Imino region of 1D ^{1}H NMR spectra of TERRA25 in 90% H_2O/10% D_2O (upper spectrum) and 15 min after transfer into 100% D_2O. The signals of the inner tetrad are protected for exchange with deuterium. Those signals fit to the imino signals assigned to the inner tetrad based on the $^{1}H,^{1}H$-NOESY.

2D NMR data to analyse effect of KCl on the structure of TERRA25

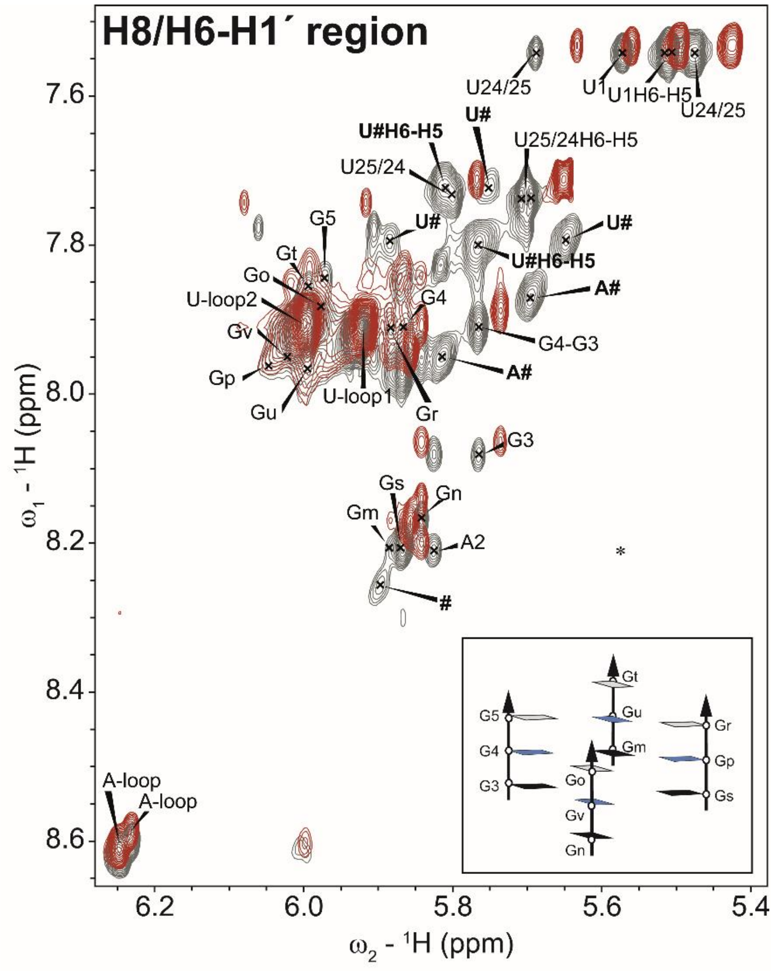

Figure S8: Overlay of 1H,1H-NOESY spectrum of TERRA25 with 4 equiv KCl (grey) and 128 equiv KCl (red). Strongest shifts show the signals of U24 and U25 as well as A2H8 and signals of the lower tetrad (G3, Gm, Gs) and of the upper tetrad (G5 and Gt). The signals of the loops (A-loop and U-loop) are nearly unaffected by KCl-addition. Most remarkably are the strong signals visible at 4 equiv KCl but not at 128 equiv KCl (assignment bold and marked with #). They are stemming from AH8 and UH6 (assignment according to 13C-HSQC spectrum, one signal could not be assigned). The increased number of signals in the NOESY spectrum show that a second and probably a third long-lived conformation is present at low KCl concentration. The conformational differences are presumably localized around the upper and lower capping nucleotides. Cross signal of U1H1´- A2H8 was detected at lower contour threshold and is marked with a star.

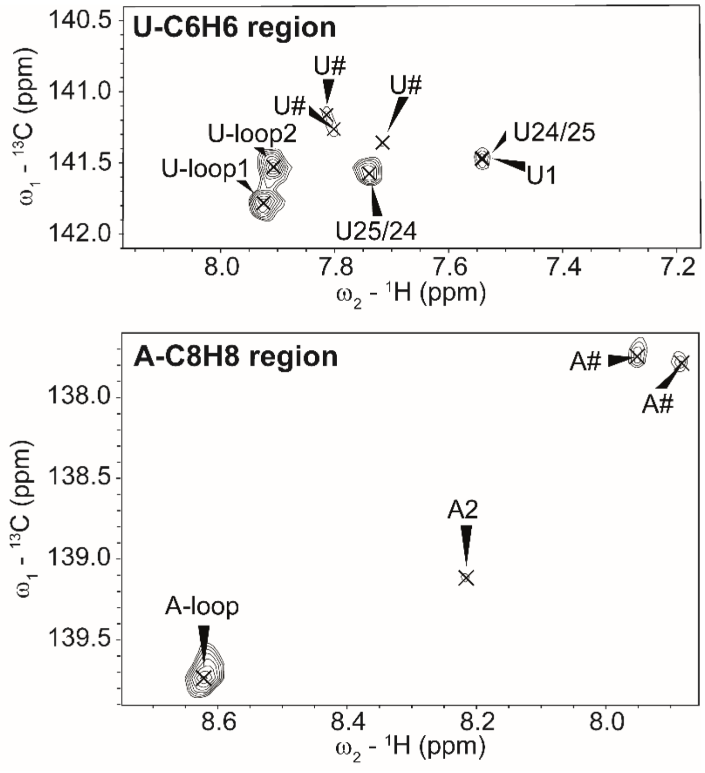

Figure S9: Extracts of ^{13}C-HSQC of TERRA25 at 4 equiv KCl to assign the strong signals in the NOESY-spectrum missing at 128 equiv KCl. These signals have been marked with #.

Analysis of the glycosidic conformation of dGMP

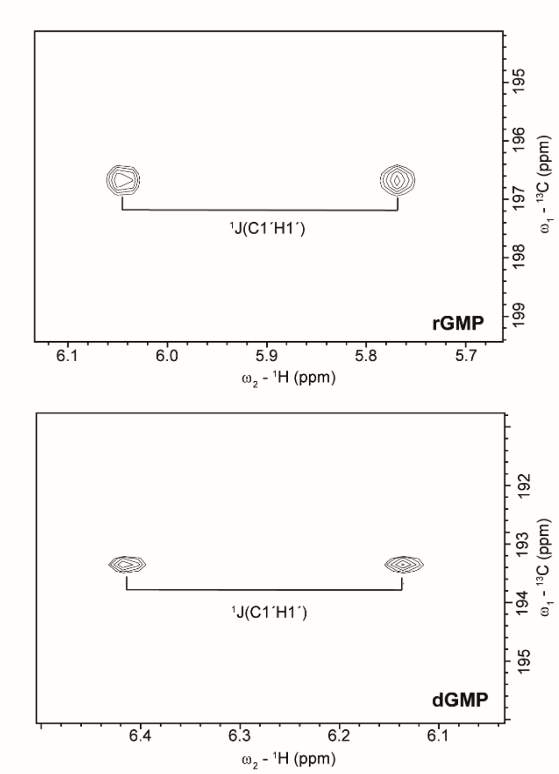

Figure S10: ^{1}H,^{13}C-HMBC of rGMP and dGMP to determine the ^1J(C1´H1´) coupling constant. The coupling constant is identical for both and has been determined to 165.9 ppm.[5]

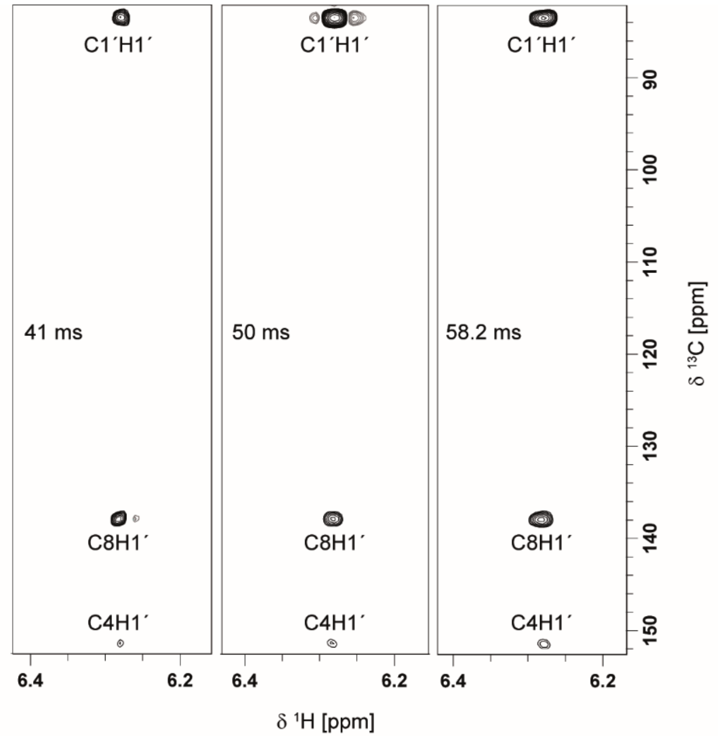

Figure S11: $^{1}H,^{13}C$-HMBC spectra of dGMP with different magnetisation transfer delays (41 ms, 50 ms and 58.2 ms). The signal intensities of C1´H1´, C4H1´ and C8H1´ have been used to calculate ^{3}J(H1´,C4) and ^{3}J(H1´,C8) according to equation 4 and 5, respectively.

Table S2: cross-peak intensities extracted from the long-range $^{1}H,^{13}C$-HMQC of dGMP recorded with respective delays delta and the resulting coupling constants.

delta [ms]	I(C1´,H1´) [abs]	I(C4,H1´) [abs]	I(C8,H1´) [abs]	^{1}J(C1´,H1´) [Hz]	^{3}J(H1´,C8) [Hz]	^{3}J(H1´,C4) [Hz]
58.2	4548940	942918	2486790	165.9	5.2	3.9
50.0	7013014	607232	1706404	165.9	3.7	2.4
41.0	833623	285989	776277	165.9	4.7	3.1
				**Mean**	**4.5**	**3.1**

Analysis of folding rates of wtTel25 determined by temperature jump-induced folding

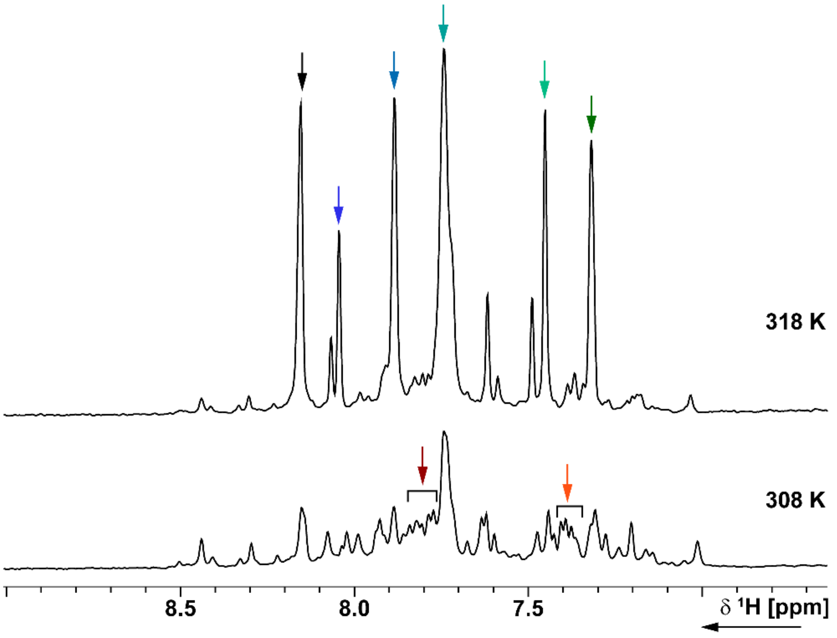

Figure S12: 1D ^{1}H spectrum of the aromatic region of wtTel25 at 318 K and 308 K. The G4 structure is nearly completely unfolded. Signals used for analysis of wtTel25 folding kinetics after temperature jump are indicated with arrows.

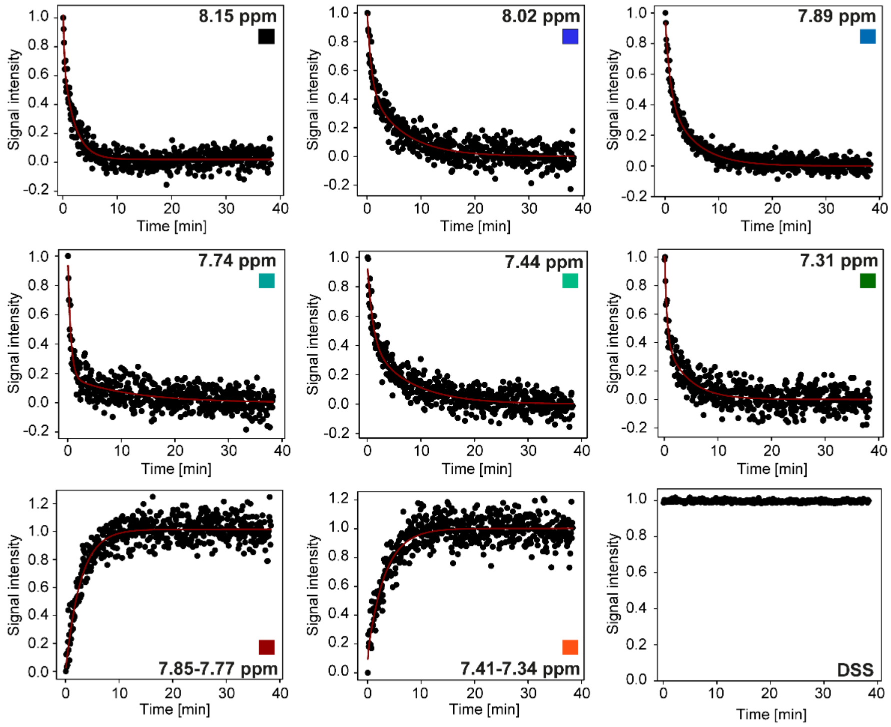

Figure S13: Kinetic traces of wtTel25 recorded at 308 K after temperature jump from 318 K that have been used to determine the observed folding rate. The DSS signal intensity is constant and shows that temperature equilibrium

of spectrometer has been reached. The colour of square indicates to which signal (marked with arrow of same colour) the kinetic trace belongs.

Table S3: mean values and standard deviation of the kinetic traces displayed in Figure S11

wtTel25	k [min^{-1}]	308 K
**Decreasing signals**	k_1	2.13 ± 1.60
	k_2	0.22 ± 0.14
**Increasing signals**	k_1	0.38 ± 0.05
	k_2	-

CD melting curves

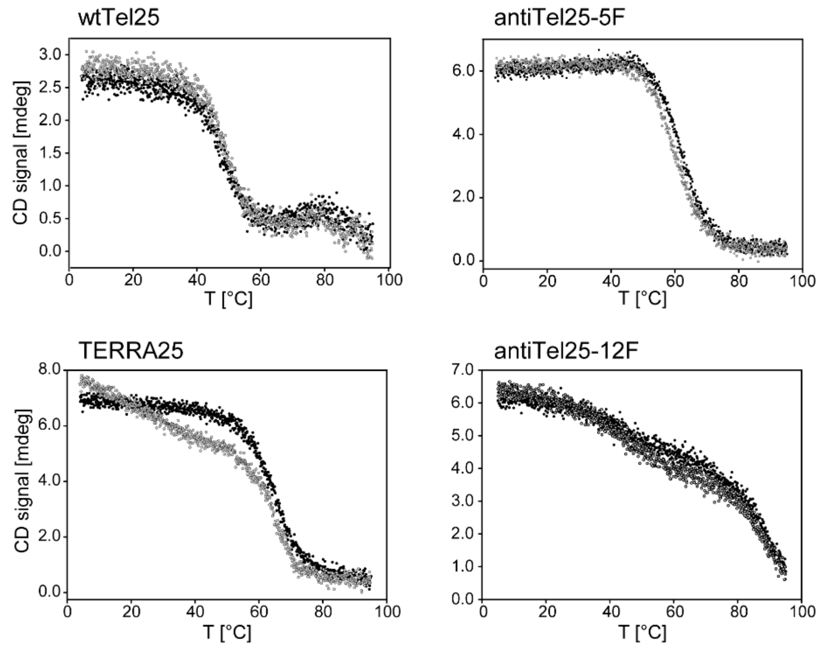

Figure S14: CD melting curves of wtTel25, antiTel25-5F, TERRA25 and antiTel25-12F. The concentration of the samples was 10 µM in 25 mM Potassium phosphate buffer pH 7.0. A heating/cooling rate of 0.5 °C/min was used. Black: melting curve, grey: annealing curve. The thermal stability increases from wtTel25 to antiTel25 that is like the TERRA25s one. The thermal stability of antiTel25-12F is further increased by 30 °C with a melting temperature of 91 °C. TERRA25 and antiTel25 have a second transition at 18 °C and 43 °C, respectively. We assume that they result from melting/formation of dimers.

Kinetic traces of the modified DNAs determined by KCl-induced folding

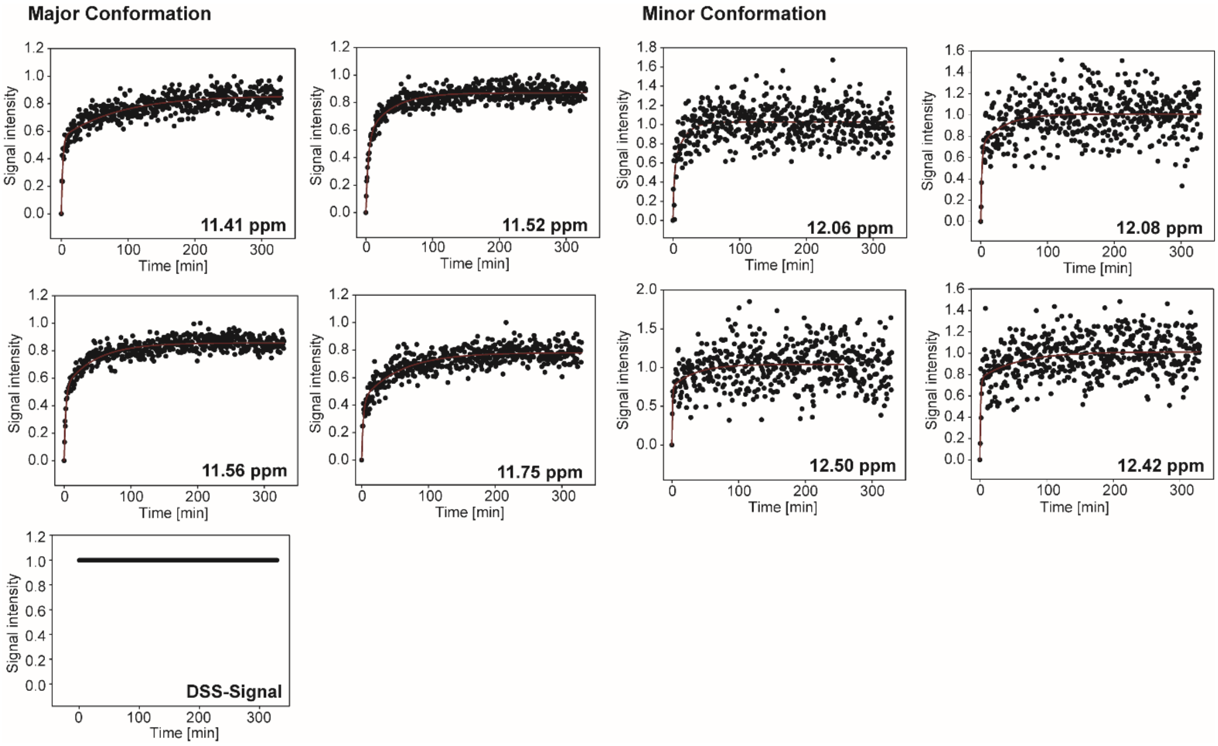

Figure S15: Kinetic traces of antiTel25-5F measured at 283 K that have been used to determine the rate constant of folding of the major and minor conformation listed in Table S1. The DSS signal served as correction factor.

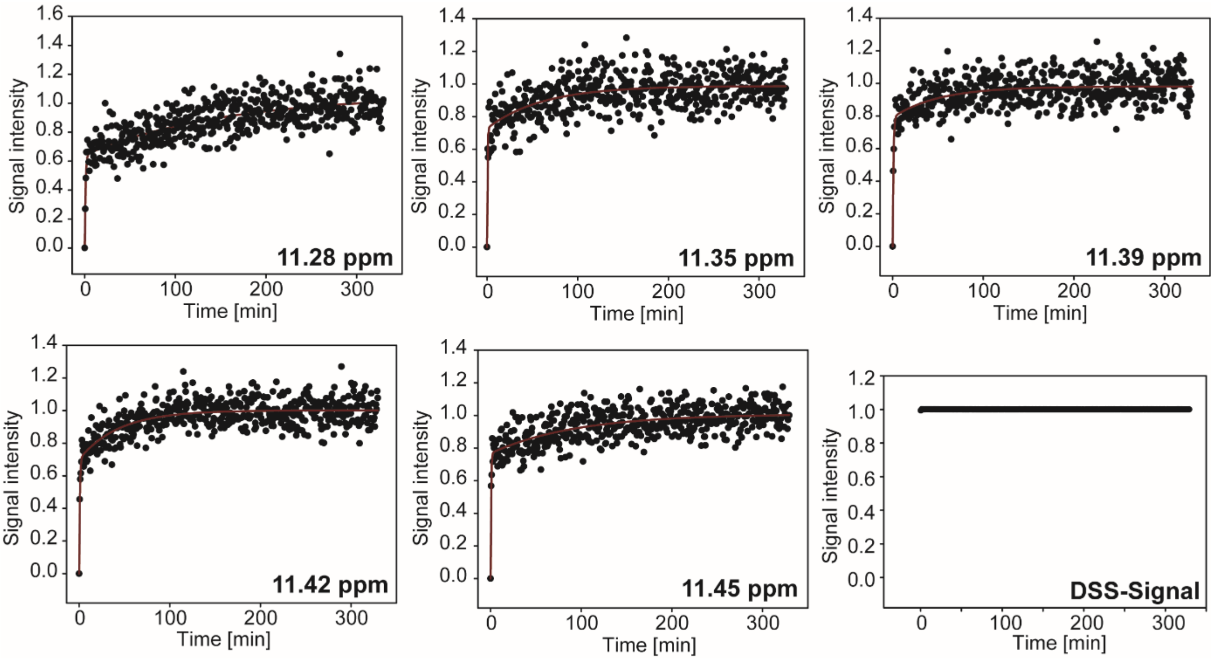

Figure S16: Kinetic traces that have been used for analysis of antiTel25-12F folding kinetics recorded at 283 K (listed in Table S1) and the kinetic trace of the DSS signal that has been used as correction factor to eliminate any changes in spectra due to the mixing procedure.

Conversion of antiTel25s minor conformation

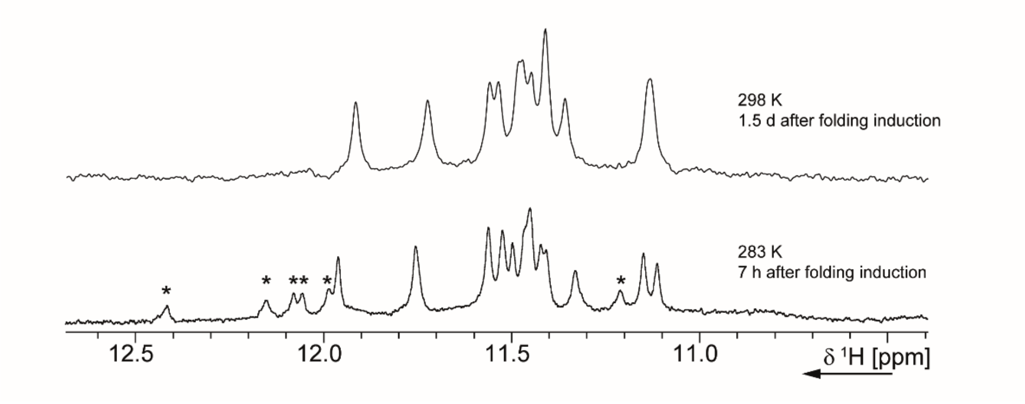

Figure S17: ^{1}H NMR spectra of antiTel25-5F 7 h after folding induction recorded at 283 K and 1.5 days after folding induction at 298 K. Signals of the kinetically trapped minor conformation are marked with stars.

Thermodynamic parameters

Table S4: Thermodynamic parameters deriving from CD-melting curves and van´t Hoff analysis and population ratio determined in 1D ^{1}H NMR spectra.

	T_M [°C]	ΔH° [kcal]	ΔS° [kcal]	ΔG° [kcal/mol] (283K)	K [major:minor]	ΔΔG [kcal] (283 K)
**wtTel25**	48	49.01	0.15	6.56	0.6:0.4	0.26
**antiTel25-5F**	61	51.62	0.15	9.17	≥ 0.92:0.08 (after 1.5 d at 298 K)	≥ 1.37
**antiTel25-12F**	91	85.27	0.23	20.18	Only one conformation	-
**TERRA25**	64	51.95	0.15	9.5	Only one conformation	-

Population of minor and minor conformation of wtTel25 and antiTel25-5F

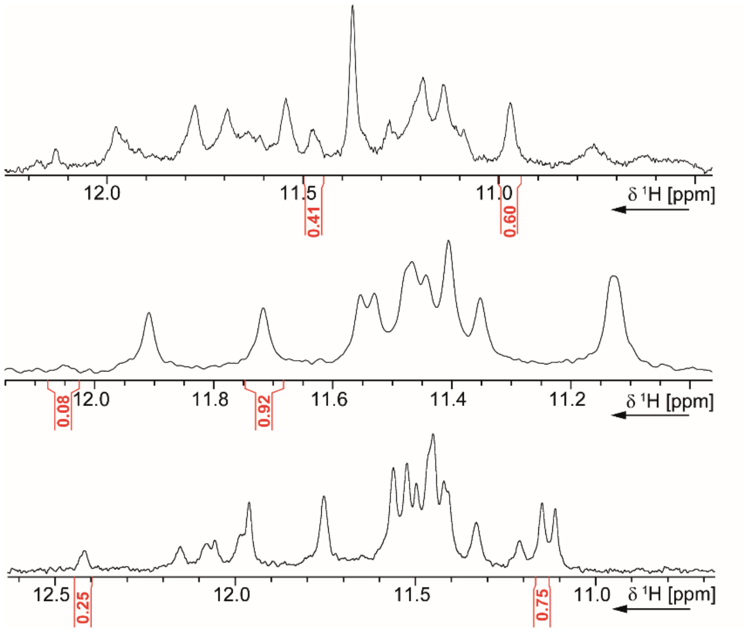

Figure S18: In ^{1}H NMR spectra recorded with excitation sculpting water suppression.[2] Imino signals clearly deriving from different folding species have been integrated with TopSpin 3.6.2 to determine the population of the two structures of wtTel25 (top spectrum) and antiTel25-5F 5 hours after folding was induced (bottom spectrum) and 1.5 days later (middle spectrum). This was used to calculate ΔΔG (ΔΔG=-RTlnk).

References

[1] V. Sklenář, A. Bax, *J. Magn. Reson.* **1987**, *74*, 469–479.
[2] T. L. Hwang, A. J. Shaka, *J. Magn. Reson. Ser. A* **1995**, *112*, 275–279.
[3] W. Lee, M. Tonelli, J. L. Markley, *Bioinformatics* **2015**, *31*, 1325–1327.
[4] P. Schanda, B. Brutscher, *J. Am. Chem. Soc.* **2005**, *127*, 8014–8015.
[5] H. Schwalbe, J. P. Marino, G. C. King, R. Wechselberger, W. Bermel, C. Griesinger, *J. Biomol. NMR* **1994**, *4*, 631–644.
[6] H. Martadinata, A. T. Phan, *J. Am. Chem. Soc.* **2009**, *131*, 2570–2578

Author Contributions

D.M. carried out the experiments and wrote the manuscript with support from I.B. and H.S. C.R. helped with NMR-related problems. I.B. and C.R. helped supervising the project. H.S. as principal investigator conceived the original idea and supervised the project.
